# Repressed Exercise-Induced Hepcidin Levels after Danggui Buxue Tang Supplementation in Male Recreational Runners

**DOI:** 10.3390/nu10091318

**Published:** 2018-09-18

**Authors:** Chih-Wei Chang, Chao-Yen Chen, Ching-Chi Yen, Yu-Tse Wu, Mei-Chich Hsu

**Affiliations:** 1 School of Pharmacy, Kaohsiung Medical University, Kaohsiung 80708, Taiwan; wxes9050304@gmail.com (C.-W.C.); date0315@hotmail.com (C.-C.Y.); ytwu@kmu.edu.tw (Y.-T.W.); 2 Center for Physical Education, Kaohsiung Medical University, Kaohsiung 80708, Taiwan; alex@kmu.edu.tw; 3 Department of Medical Research, Kaohsiung Medical University Hospital, Kaohsiung 80756, Taiwan; 4 Department of Sports Medicine, Kaohsiung Medical University, Kaohsiung 80708, Taiwan

**Keywords:** iron deficiency, exercise performance, *Angelica sinensis*, *Astragalus membranaceus*, traditional Chinese medicine, sports nutrition

## Abstract

This study was to investigate the protective and recovery effects of Danggui Buxue Tang (DBT) supplementation on exercise performance, hepcidin, iron status, and other related biochemical parameters after being challenged by a single bout of intense aerobic exercise. A total of 36 recreationally active males were pair-matched and randomly assigned to receive DBT or a placebo for 11 days, while using clusters based on their aerobic capacities. On the eighth day of the supplementation, the participants performed a 13-km run with maximal effort. Blood and urine samples were collected and analysed before treatment (Pre-Tre) and immediately after (Post-Ex), 24 h after (24-h Rec), and 72 h after (72-h Rec) the run. DBT supplementation dramatically shortened the finish times by 14.0% (12.3 min) when compared with that in the placebo group. Significant group × time effects were observed in serum hepcidin and iron levels. DBT supplementation repressed hepcidin levels at Post-Ex and 24-h Rec, thereby causing a significant increase in iron levels by 63.3% and 31.4% at Post-Ex and 72-h Rec, respectively. However, DBT supplementation had no significant anti-inflammatory or haemolysis-preventative effects. Short-term DBT supplementation shortened the running time and repressed exercise-induced hepcidin levels, thereby boosting iron levels and accelerating iron homeostasis during recovery.

## 1. Introduction

Iron is an essential trace element that plays a fundamental role in haemoglobin synthesis, oxygen delivery, and electron transport chains that are involved in physical exercise [1]. The prevalence of iron deficiency in athletes is notably high (up to 30%) [2,3,4]. Exercise-related iron deficiency has been reported to be associated with several well-established mechanisms, such as gastrointestinal bleeding, haematuria, sweating, and haemolysis [5]. However, post-exercise hepcidin response was previously demonstrated as the most critical mechanism leading to a degraded iron status [6].

Hepcidin is the principal hormone responsible for iron homeostasis, which maintains the absorption of dietary iron and the distribution of iron among body tissues [7]. Exercise-induced hepcidin elevation degrades the iron transporters—ferroportin [8] and the divalent metal transporter—resulting in blocked duodenal iron absorption, cellular iron efflux, and the ability to recycle iron from macrophages [1]. Several factors that are involved during exercise have been highlighted to attribute to the regulation of hepcidin expression, for instance, circulating iron, oxygen disturbances, and inflammatory responses, particularly via the IL-6/STAT3 signalling pathway; consequently, these lead to the peak elevation in serum hepcidin levels generally being observed at 3-h post-exercise [9]. Studies have demonstrated the performance of numerous physical activities could impact the hepcidin response, namely, marathons [10], 10-km runs [11], military training [12], basketball games [13], and rowing [14]. Foot-strike or weight-bearing exercise, such as running, has been demonstrated to produce higher hepcidin levels than non-weight-bearing exercise [15] and cause haemolysis [16].

Regardless of anaemia, iron deficiency impairs muscle function, limits work capacity [17], and diminishes aerobic performance [18]. Ferrous salts that are used in oral therapy are the most common medication for the treatment of iron deficiency. However, their gastrointestinal side effects (e.g., nausea, flatulence, abdominal pain, diarrhoea, constipation, and black or tarry stools) have been a principal concern for decades [19]. Alleviating iron absorption and utilisation from food sources while using additional supplementation is one alternative approach that has been considered.

Danggui Buxue Tang (DBT), which is a familiar Chinese herbal decoction, was first recorded in Nei Wai Shang Bian Huo Lun during the Jin dynasty (1247 BC) and it was used to treat ailments in women. DBT contains the roots of *Astragalus membranaceus* (Fisch.) Bge. var. *mongholicus* (Bge.) Hsiao (denoted AM) and the roots of *Angelica sinensis* (Oliv) Diels (denoted AS) at a respective weight ratio of 5:1. According to traditional Chinese medicine, AM can increase a person’s “qi” (vital energy), whereas AS can nourish a person’s “blood” (body circulation). Because of the safety of these ingredients, the components of DBT are commonly used in Chinese cuisine or as a dietary supplement.

A series of comprehensive studies conducted by Zhang et al. have highlighted that the polysaccharides present in AS can suppress hepcidin and its signal pathway [20,21,22,23]. An earlier study confirmed that DBT significantly increased iron absorption when being co-administrated with ferrous sulfate in iron-deficient anaemic rats [24]. In addition, previous studies published by our research group revealed that AM and AS have promising effects for improving exercise performance, ameliorating fatigue by reducing serum lactate and ammonia levels, as well as increasing muscle glycogen content and even promoting hypertrophy [25,26,27,28]. DBT may function in the normalisation of the levels of proinflammatory cytokines, tumour necrosis factor (TNF), and interleukin (IL), and in the elevation of endurance capacity in rats [29]. To date, there is a lack of clinical evidence regarding the effect of DBT on iron regulation and exercise performance.

The objective of this study was to investigate the protective and recovery effects of short-term DBT supplementation on exercise performance, hepcidin, iron status, and other related biochemical parameters after recruited male recreational runners performed a single bout of aerobic exercise (13-km run).

## 2. Methods

### 2.1. Preparation of DBT and Placebo

The DBT granule preparation was bought from Kaiser Pharmaceutical Co., Ltd. (Tainan, Taiwan), which has a certification of Good Manufacturing Practice. In the product line of the DBT granule preparation, raw plant materials, namely AM and AS, was obtained from Mongolia, China and Gansu, China, respectively. The origin of the herb first underwent identification and authentication by qualified experts through the company’s protocol, including macroscopic inspection, microscopic examination, and thin layer chromatography. The best herbs were selected, cleaned, sliced, and extracted with the traditional method. The decoction liquid evaporated in low-temperature vacuum and then spray-dried to form granules. To establish the safety, the product was also screened for bio-contaminants, pesticides, and heavy metals.

The daily consumption used in this study was 7.5 g of DBT, composed of 5.0 g of plant extract (prepared using 25.0 g of crude AM and 5.0 g of crude AS) and 2.5 g of cornstarch. In the placebo, the 5.0-g plant extract was replaced by carboxymethyl cellulose. Both DBT and placebo were then encapsulated into brown gelatin capsules (Dah Feng Capsule, Taichung, Taiwan) a week before the participants began to consume them. The DBT and placebo capsules were identical in their appearance, colour, and weight.

### 2.2. Phytochemical Analysis of DBT

#### 2.2.1. Chemicals and Reagents

Anhydrous glucose and rutin were purchased from Sigma-Aldrich (St. Louis, MO, USA). Astragaloside IV was purchased from the National Institutes for Food and Drug Control (Beijing, China). In addition, ferulic acid was purchased from Acros Organics (Geel, Belgium) and ligustilide was purchased from Grand Chemical (Bangkok, Thailand). *n*-Butylidenephthalide was purchased from Alfa Aesar (Ward Hill, MA, USA). Purified water was prepared while using the Barnstead Easypure II water purification system (Thermo Scientific, Waltham, MA, USA). Analytical-grade acetonitrile was obtained from Honeywell (Morris Plains, NJ, USA), methanol was obtained from Avantor Performance Materials (Center Valley, PA, USA), and acetic acid and phosphoric acid were obtained from Merck (Darmstadt, Germany) (Table 1).

#### 2.2.2. Apparatus

A spectrophotometer (PRO-7990, Prema, Taipei, Taiwan) was used for the determination of polysaccharides and total flavonoids. A high-performance liquid chromatography (HPLC) system comprising a four-channel gradient delivery system (600, Waters, Milford, MA, USA), an autosampler (717 Plus, Waters), a column heater (U-620, Sugai, Wakayama, Japan), an evaporative light scattering detector (ELSD; Sedex 75, Sedere, Alfortville, France), and a photodiode array (PDA) detector (2996, Waters) were used for the determination of astragaloside IV, ferulic acid, ligustilide, and *n*-butylidenephthalide. A Cosmosil 5C18-MS-II column (250 × 4.6 mm, i.d. 5 μm; Nacalai Tesque, Kyoto, Japan) with a Lichrospher RP-18 end-capped guard column (10 × 4.0 mm, i.d. 5 μm; Merck, Darmstadt, Germany) was used in the HPLC separation.

#### 2.2.3. Determination of Polysaccharides and Total Flavonoids

The polysaccharide and the total flavonoid contents of DBT were determined while using spectrophotometric methods according to the Pharmacopoeia of the People’s Republic of China [30], wherein anhydrous glucose and rutin served as the standards, respectively.

#### 2.2.4. Determination of Astragaloside IV

A DBT sample (5.0 g) was subjected to ultrasonic-assisted extraction using 50 mL of methanol at 25 °C for 30 min. After centrifugation, concentration, and filtration, the solution was injected into HPLC-ELSD for the determination of astragaloside IV. Gradient elution was used with 0.1% acetic acid as solvent A, acetonitrile as solvent B, and pure water as solvent C. The gradient program was as follows: 0–20 min, 83–65% A, 17–35% B, and 0–0% C; 20–45 min, 65–0% A, 35–65% B, and 0–35% C; 45–50 min, 0–0% A, 65–17% B, and 35–83% C; and 50–55 min, 0–83% A, 17–17% B, and 83–0% C. The flow rate was set at 1.0 mL·min^−1^ and the injection volume was 20 μL. The column temperature was set at 35 °C. The ELSD drift tube temperature was set at 80 °C and the pressure was set at 2.3 bar.

#### 2.2.5. Determination of Ferulic Acid, Ligustilide, and *n*-Butylidenephthalide

A DBT sample (1.0 g) was subjected to ultrasonic-assisted extraction using 20 mL of methanol:water (70:30, *v*/*v*) at 25 °C for 15 min, followed by shaking at 160 rpm and 40 °C for 20 min. After centrifugation and filtration, the solution was injected into the HPLC-PDA detector for the determination of ferulic acid, ligustilide, and *n*-butylidenephthalide. Gradient elution was used with 0.085% phosphoric acid as solvent A and acetonitrile as solvent B. The gradient program was as follows: 0–10 min, 90–85% A; 10–20 min, 85–80% A; 20–30 min, 80–60% A; 30–55 min, 60–35% A; 55–65 min, 60–0% A; and, 65–80 min, 0–90% A. The flow rate was set at 1.0 mL·min^−1^ and the injection volume was 40 μL. The column temperature was set at 35 °C, and the detection wavelength was set at 320 nm.

### 2.3. Clinical Study

#### 2.3.1. Ethics Approval

The trial’s protocol was reviewed and approved by the Institutional Review Board of Kaohsiung Medical University, Chung-Ho Memorial Hospital, Kaohsiung, Taiwan (IRB Number: KMUHIRB-F(I)-20160078), and registered in ClinicalTrials.gov (Identifiers: NCT02996786). All of the participants signed informed consent forms before entering into the trial, and the trial was conducted in accordance with the Declaration of Helsinki and approved guidelines.

#### 2.3.2. Participants

Inclusion criteria were as follows: age 20–30 years, being healthy, and being a recreationally active male who had experience in completing runs of at least 10 km but who had never performed a 42-km marathon run. Exclusion criteria included having anaemia (Hb < 13 g·dL^−1^) or any other diseases, smoking, engaging in regular alcohol consumption, and using ergogenic aids or medication. Each participant completed the Physical Activity Readiness Questionnaire (PAR-Q) to ensure their safety before they undertook the exercise activity; if the answer was YES to one or more questions, the participant was ruled out.

#### 2.3.3. Experimental Design

**Figure 1:**
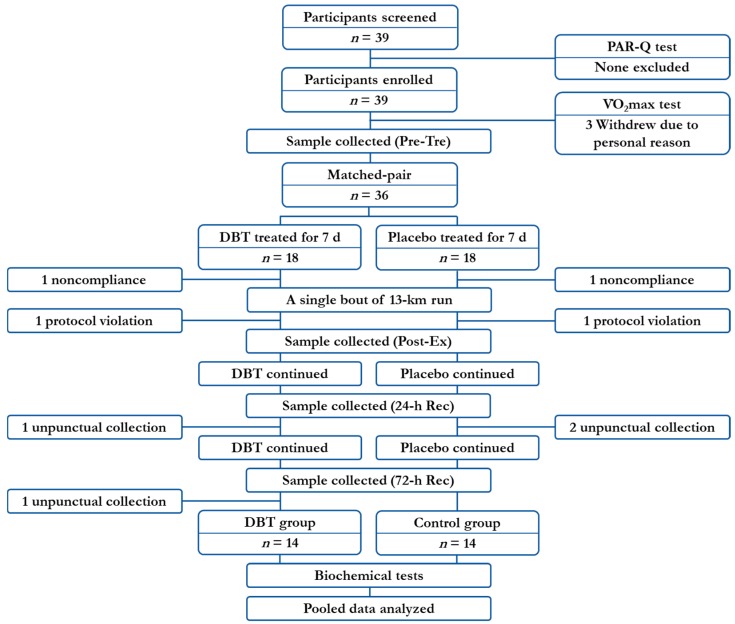
Flowchart of participant enrollment, allocation, and treatment.

The participant recruitment and baseline data collection started from 13 October 2016, the run was performed on 10 December 2016, and was followed by several sample collections until the end of the study on 13 December 2016. The study was designed as a matched-pair, cluster-randomised controlled trial. A total of 39 participants were screened, 36 participants received supplementation, and 28 participants were eligible for data analysis (Figure 1). The participants were first distributed into the following three clusters based on their individual maximal oxygen consumption (representing their aerobic capacity): (i) <50.0, (ii) 50.0–64.9, and (iii) >65.0 mL·kg^−1^·min^−1^. They were then randomly assigned into one of two supplementation groups, namely (a) control group (*n* = 14) and (b) DBT group (*n* = 14), so that there were equal numbers of control and DBT participants in each cluster. The DBT group voluntarily ingested 7.5 g·day^−1^ of DBT (BID PC) for 11 days, whereas the control group received the placebo in the same manner. The exercise training characteristics (including the type, frequency, and intensity of the exercise) of each volunteer was documented at the screening test to ensure that they were recreationally active. During the study period, the participants were strictly informed not to perform any extra high-intensity physical practice apart from their original habits. The participants were also asked to maintain their regular diet and refrain from consuming any supplements or extra antioxidant-rich foods that they did not normally consume (e.g., tea, coffee, fruit, and vegetables). All of the participants understood the study regulations and consented to comply with the protocol.

On the eighth day of the supplementation, the participants performed a 13-km run by running five laps (2.6 km·lap^−1^) on the sidewalk around the Kaohsiung Museum of Fine Arts, Kaohsiung, Taiwan, with maximum effort. The temperature was 21.7–23.7 °C, relative humidity was 66–78%, wind speed was 0.9–1.6 m·s^−1^, and air pressure was 1011.5–1012.8 hPa. A soy bar (SOYJOY, Otsuka Pharmaceutical, Tokyo, Japan) alongside with a DBT or a placebo were administered immediately prior to the run began. Adequate water, sports drinks (Pocari Sweat, Otsuka Pharmaceutical), and bananas were supplied during the run to maintain their energy levels. Their finish times were recorded.

Blood (17 mL) and urine (10 mL) samples were collected before treatment (Pre-Tre), immediately after (Post-Ex), 24-h after (24-h Rec), and 72-h after (72-h Rec) the 13-km run. The timeframe of the specimen collections was consistent (between 9:00 and 10:00 a.m.). The whole blood samples were collected from the antecubital vein and then split into three tubes containing one of clot activator, K_2_EDTA, or heparin (BD, Franklin Lakes, NJ, USA). Serum and plasma samples were obtained from the tubes containing the clot activator and heparin, respectively, after centrifuging them at 3000 rpm for 10 min, and these samples were then subjected to further biochemical analysis.

### 2.4. Measurement of Maximal Oxygen Consumption

Maximal oxygen consumption (VO_2_max), the most valid measure of the functional capacity of the cardiorespiratory system, reflects the capacity of the heart, lungs, and blood to deliver oxygen to the working muscle during dynamic exercise involving large muscle mass [31].

The participants performed a graded exercise test—the Bruce protocol [32]—on a motor-driven treadmill (h/p/cosmos, Nussdorf-Traunstein, Germany), and their oxygen consumption, heart rate, and rating of perceived exertion (RPE) were recorded throughout. The test began at 1.7 mph and a 10% incline, and the speed and the incline of the treadmill were, respectively, increased by 0.8 or 0.9 mph and 2% every 3 min. Gas exchanges were measured breath-by-breath while using a gas analyser (K4b2, Cosmed, Rome, Italy) and subsequently averaged over 30-s intervals. Heart rate was monitored while using a heart rate monitor (Polar Electro, Kempele, Finland).

VO_2_max was confirmed by the satisfaction of at least three of the following criteria [33]: (i) a plateau in oxygen uptake defined as no expected increase of more than 150 mL·min^−1^, despite an increase in power output; (ii) a respiratory exchange ratio >1.1; (iii) a heart rate ±10% of age-predicted maximal heart rate (210–0.65 × age); and, (iv) an RPE >17 on Borg’s 6–20 scale [34].

### 2.5. Biochemical Analysis of Blood and Urine

All biochemical analyses were performed in accordance with the manufacturers’ instructions. Complete blood counts, including platelet, white blood cell (WBC), red blood cell (RBC), haemoglobin (Hb), haematocrit (HCT), mean corpuscular volume (MCV), mean corpuscular haemoglobin (MCH), and mean corpuscular haemoglobin concentration (MCHC), were assayed using the CELL-DYN Ruby Hematology System (Abbott Laboratories, Chicago, IL, USA) with multiangle polarized scatter separation laser technology. The levels of serum iron, ferritin, transferrin, haptoglobin, lactic dehydrogenase (LDH), aspartate aminotransferase (AST), alanine aminotransferase (ALT), and plasma ammonia were assayed while using a TBA-c16000 automatic analyser (Toshiba, Tochigi, Japan). Iron level was measured using the colourimetric method, and ferritin level was measured while using the latex agglutination method. In addition, transferrin and haptoglobin levels were measured using immunoturbidimetric assay. The levels of LDH, AST, and ALT were measured while using the Japan Society of Clinical Chemistry standard method. Ammonia level was measured using the glutamate dehydrogenase method. The levels of serum malondialdehyde (MDA), superoxide dismutase (SOD), catalase (CAT), and plasma glutathione peroxidase (GPx) were assayed using Cayman assay kits (Ann Arbor, MI, USA), with the item numbers 10009055, 706002, 707002, and 703102, respectively. Furthermore, serum hepcidin-25 was assayed using DRG ELISA kits (Marburg, Germany), with the item number EIA-5782. Serum TNF-α and IL-6 levels were assayed using R & D High Sensitivity ELISA kits (Minneapolis, MN, USA), with the item numbers HSTA00D and HS600B, respectively. Urine occult blood (OB) was assayed using the AUTION MAX AX-4030 Urinalysis System (ARKRAY, Kyoto, Japan) with the dual-wavelength reflectance method.

### 2.6. Statistical Analysis

SPSS 19.0 (International Business Machines Corporation, Armonk, NY, USA) was used for statistical analyses. Data are expressed as mean (±standard deviation). Differences in age, height, weight, maximal heart rate (HRmax), VO_2_max, 13-km finish time, and basal iron status between the two groups were analysed while using the independent *t*-test. The relationship between VO_2_max and finish time was analysed in each group using Spearman’s correlation. The effect of group and finish time interaction (group × time) on the blood biochemical parameters was analysed using two-way repeated measures analysis of variance (ANOVA). To minimise the violation of the assumption of homogeneity of variance, the Greenhouse–Geisser correction was used when Mauchly’s test of sphericity was violated. The significance of group × time interaction indicated that the levels of the parameters of the groups were changing over time, but in different ways (not parallel). The paired *t*-test was used for further determination of the differences between Pre-Tre and other time points in each independent group. A *p* value < 0.05 was considered as statistically significant.

## 3. Results

### 3.1. Phytochemical Properties of DBT

**Figure 2:**
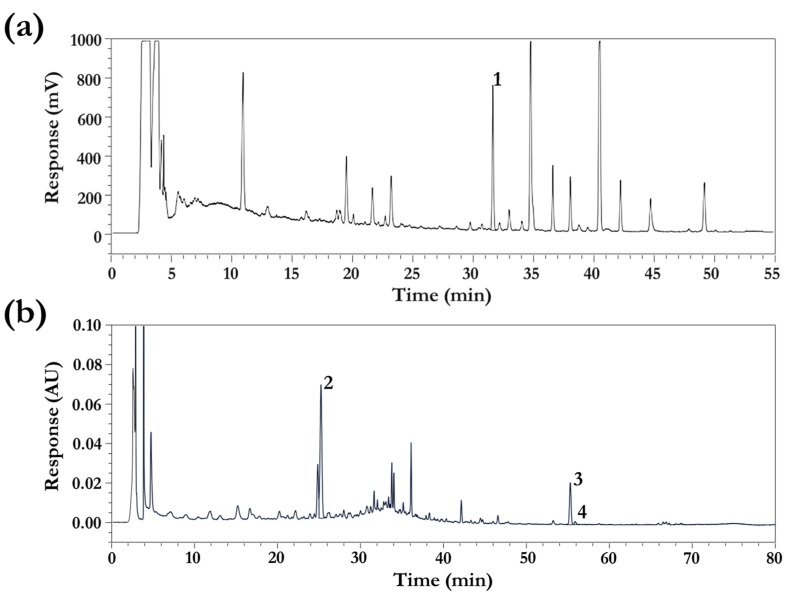
The chromatogram of DBT analysed using (**a**) HPLC-ELSD and (**b**) HPLC-PDA. ^1^ astragaloside IV, ^2^ ferulic acid, ^3^ ligustilide, ^4^ *n*-butylidenephthalide.

The bioactive constituents of DBT—including astragaloside IV, flavonoids, and polysaccharides from AM and ferulic acid, ligustilide, *n*-butylidenephthalide, and polysaccharides from AS—were qualitatively and quantitatively confirmed. The polysaccharide and total flavonoid contents in DBT were 284.35 and 4.55 mg·g^−1^, respectively. The contents of astragaloside IV, ferulic acid, ligustilide, and *n*-butylidenephthalide in DBT were 0.11, 0.12, 0.05, and 0.01 mg·g^−1^, respectively. The HPLC chromatogram of DBT is depicted in Figure 2.

### 3.2. Characteristics and Exercise Performance of Participants

**Figure 3:**
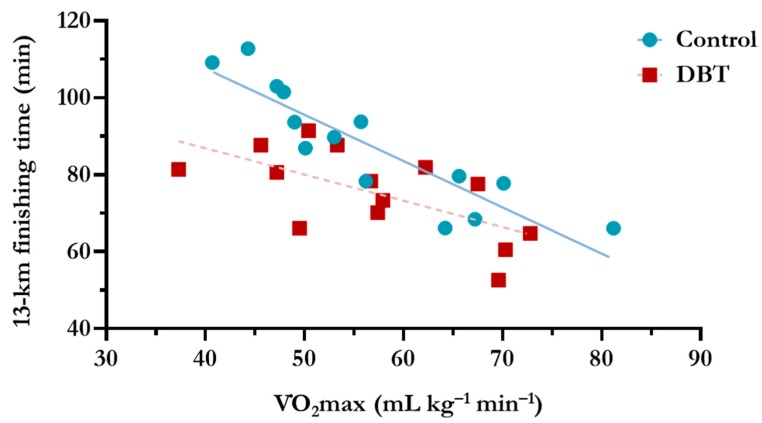
Relationship between VO_2_max and finish time in each group as analysed using Spearman’s correlation (*r* = −0.925, *p* < 0.001, slope = −1.203 in control group; *r* = −0.649, *p* < 0.05, slope = −0.682 in DBT group).

There were no significant differences in age, height, weight, and HRmax between the groups at baseline (*p* > 0.05) (Table 2). The baseline VO_2_max of each group was also not different (*p* > 0.05); however, the 13-km finish time of the DBT group was significantly shorter than that of the control group by 14.0% (12.3 min; *p* = 0.024). As illustrated in Figure 3, a strong correlation was discovered between VO_2_max and 13-km finish time in the control group (*r* = −0.925, *p* < 0.001), confirming that the baseline VO_2_max highly correlated with the aerobic performance of each untreated participant; however, a relatively weak correlation was observed in the DBT group (*r* = −0.649, *p* = 0.012). Most of the data points for the DBT group were plotted below the control group’s linear regression line, indicating that the DBT supplementation resulted in a shortened finish time and an improved exercise performance. Remarkably, the slope of the linear regression line was more horizontal for the DBT group (−0.682) than that for the control group (−1.203), which highlighted that a larger improvement in exercise capacity was observed in the participants with a lower baseline VO_2_ max.

### 3.3. Iron Status and Haptoglobin Levels

**Figure 4:**
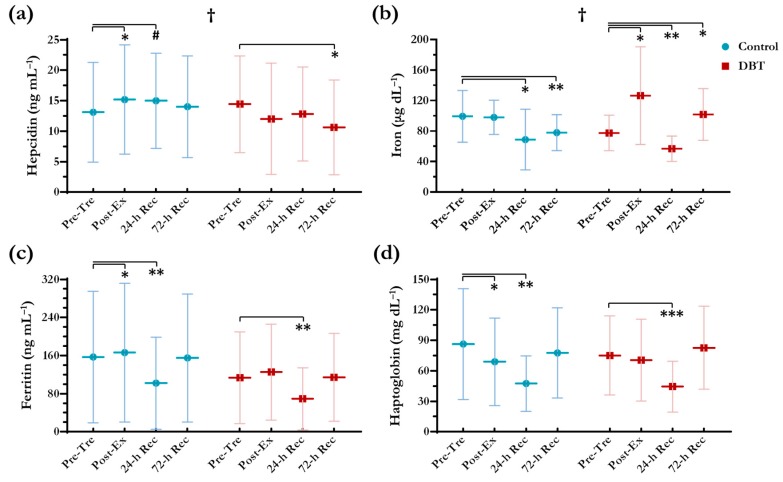
Changes in serum levels of (**a**) hepcidin, (**b**) iron, (**c**) ferritin, and (**d**) haptoglobin. ^†^ Significant group × time effect (*p* < 0.05) analysed using two-way repeated measures ANOVA. ^#^ *p* = 0.050, * *p* < 0.050, ** *p* < 0.010, *** *p* < 0.001 as compared with Pre-Tre in each independent group using paired *t*-test.

No differences (*p* < 0.05) were found between the two groups in basal iron status analysed by independent *t*-test (Table 3). A significant group × time effect was observed in the participants’ hepcidin levels (*p* < 0.05; Figure 4a). Induced by the exercise, the hepcidin levels of the control group were increased by 15.9% and 14.4% at Post-Ex (*p* = 0.035) and 24-h Rec (*p* = 0.05), respectively, and then recovered to the baseline level at 72-h Rec. However, in the DBT group, we did not observe significant changes in the hepcidin levels at Post-Ex and 24-h Rec as compared with those at Pre-Tre (*p* > 0.05); furthermore, the hepcidin levels were decreased by 26.2% at 72-h Rec (*p* = 0.012). Remarkably, a significant group × time effect was also detected in the participants’ iron levels (*p* < 0.05) (Figure 4b). After performing the exercise, the control group’s iron levels remained unchanged at Post-Ex (*p* > 0.05), but they were markedly decreased by 30.7% at 24-h Rec (*p* = 0.033) and still did not recover at 72-h Rec (*p* = 0.008). DBT supplementation for 7 d led to a significant increase of 63.3% in iron levels at Post-Ex (*p* = 0.019); in addition, the relatively large standard deviations suggested that some of the DBT group participants experienced remarkable amelioration in iron absorption and metabolism. DBT supplementation could not prevent the decrease in iron levels at 24-h Rec (26.7%; *p* = 0.004); however, an immediate recovery was observed at 72-h Rec (*p* = 0.025).

No significant group × time effect was identified in ferritin levels (*p* > 0.05) (Figure 4c). The ferritin levels changed over time, but these changes were not different between the groups. The ferritin levels were significantly decreased at 24-h Rec in both groups (*p* < 0.01). Furthermore, the haptoglobin levels were significantly decreased at 24-h Rec in both of the groups (*p* < 0.01) (Figure 4d), signifying the occurrence of haemolysis.

### 3.4. Oxidative Stress and Antioxidant Activities

**Figure 5:**
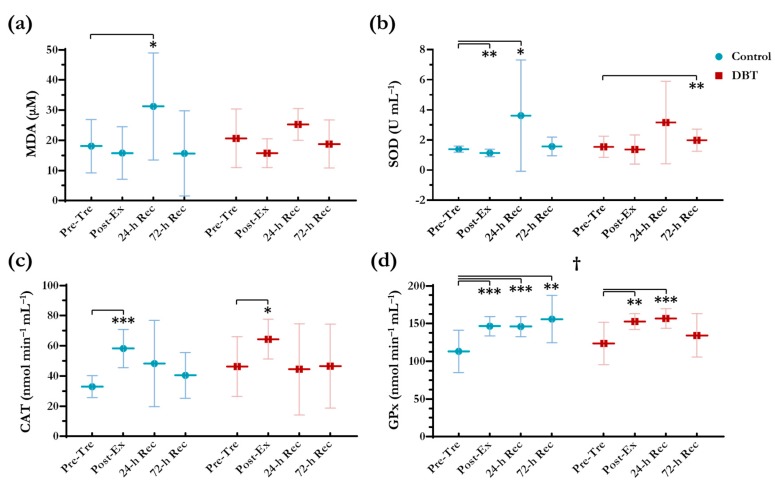
Changes in serum levels of (**a**) malondialdehyde (MDA), (**b**) superoxide dismutase (SOD), (**c**) catalase (CAT), and plasma levels of (**d**) glutathione peroxidase (GPx). **^†^** Significant group × time effect (*p* < 0.05) analysed using two-way repeated measures ANOVA. * *p* < 0.050, ** *p* < 0.010, *** *p* < 0.001 as compared with Pre-Tre in each independent group using paired *t*-test.

As illustrated in Figure 5, a significant increase was observed in MDA levels, a product of lipid peroxidation, of 72.7% at 24-h Rec in the control group (*p* = 0.032), whereas the MDA levels increased non-significantly by 22.5% in the DBT group (*p* = 0.151). No significant group × time effect was determined for the MDA, SOD, and CAT levels. The trends in these enzyme responses did not differ between the two groups. However, a significant group × time effect was detected in the GPx responses. The GPx levels increased at Post-Ex, 24-h Rec, and 72-h Rec in the control group (*p* < 0.01), but there was a recovery at 72-h Rec in the DBT group (*p* > 0.05).

### 3.5. Complete Blood Counts, Inflammatory and Metabolic Markers

Complete blood counts and concentrations of inflammatory and metabolic markers are listed in Table 4. After the exercise challenge, the WBC and platelet concentrations were significantly increased at Post-Ex in both groups (*p* < 0.01), whereas RBC, HCT, and MCH levels were significantly decreased at 24-h Rec and 72-h Rec in both groups (*p* < 0.05). The Hb levels were also significantly decreased at 24-h Rec and 72-h Rec in the DBT group (*p* < 0.05), and significant decreases in MCV levels at Post-Ex, 24-h Rec, and 72-h Rec were observed in the control group as well (*p* < 0.01). The responses of the inflammatory markers, namely TNF-α and IL-6, did not differ between the two groups. The levels of these inflammatory markers were significantly increased at Post-Ex and 24-h Rec (*p* < 0.0001) and then returned to the baseline level at 72-h Rec (*p* > 0.05). There were also no differences in the trends in the metabolic marker responses (AST, ALT, LDH, and ammonia) between the two groups.

### 3.6. Urine OB

The urine OB results are presented in Table 5. In the control group, three positive results were observed at Post-Ex (two of +/− and one of +) and two positive results were observed at 24-h Rec; in the DBT group, there were three positive results at Post-Ex (two of ++ and one of +++) and no positive results at 24-h Rec. No positive results were identified at 72-h Rec in either group. These results suggested that DBT supplementation did not prevent the immediate production of haematuria after exercise.

## 4. Discussion

DBT, which is an ancient herbal decoction, has gained considerable attention in recent years because of the general belief that traditional DBT recipes have synergic and therapeutic effects. The theory of traditional Chinese medicine states that “qi” should hold “blood,” while “blood” should carry “qi.” The combination of “qi” and “blood” is vital for maintaining bodily health. Consequently, the synergic effects of AM and AS in DBT have been verified in previous studies. The permeability and solubility of formononetin and calycosin from AM—the most predominant compounds with estrogenic, erythropoietic, and osteogenic functions [35]—were found to be enhanced by AS and its derived ferulic acid [36,37]. However, the oils from AS (e.g., ligustilide) have been demonstrated to suppress the biological properties of DBT [38,39].

Previous studies have revealed numerous beneficial effects of DBT, such as renoprotective [40], hepatoprotective [41,42], cardioprotective [43], pulmonary protective [44,45], anti-inflammatory [46], immunostimulant [47,48], antioxidant [49,50], and erythropoietic [51,52] properties. However, the majority of these studies were in vitro tests and used animal models. Haines et al. [53] and Wang et al. [54] had investigated the beneficial effects of DBT on menopausal symptoms in Chinese women, and Du et al. [55] had explored the effect of DBT on the immune function in patients with non-small-cell lung cancer. Nonetheless, the published clinical evidence regarding the effects of DBT is relatively scant.

The daily dose of DBT concentrate that we administered to the participants was prepared using 25 g of AM and 5 g of AS. According to the Taiwan Herbal Pharmacopeia [56], such a dose has been recorded in the ancient book Nei Wai Shang Bian Huo Lun, with the effects of alleviating qi weakness, blood deficiency, and fatigue. A meta-analysis study [57] reviewed the current clinical practices and the safety of administering DBT in renal anaemic patients of either gender. In most of the included studies, the patients were aged 40–60 years and they were administered daily AM and AS at the respective weights of 30 g and 6 g or more. In general, AM and AS exhibit a wide margin of safety [58], which might explain the lack of an absolute dose range or the ratio of this folk formula for better treatment. Hence, although the literature lacks the clinical evidence regarding the relationship between the dose of DBT and its efficacy, we recruited healthy male volunteers aged 20–30 years and chose to administer DBT with the original dose that has been mentioned since ancient times to minimise the possible adverse effects and individual differences.

We investigated the effects of DBT in male adults who performed a 13-km run. Female adults are more likely to experience iron depletion; however, variables, such as menstruation-related hormonal changes and blood loss, should be taken into consideration. A possible way to eliminate the variations is to recruit the female oral contraceptive pill (OCP) users. Sim et al. [59] suggested that exercise that was performed during the different phases of a monophasic OCP regulated cycle did not alter exercise-induced IL-6 or hepcidin production. Regarding the distance of the run, it has been well documented that blood loss occurs particularly at distances that exceed 10 km [60], and 13 km is a common distance for runners. We also propose that running on the road rather than on a treadmill led to greater mechanical trauma caused by the ground impact involved.

For an objective evaluation of the efficacy of the supplementation, a matched-pair, cluster-randomised design was applied, which was based on individual VO_2_max level. No difference was found in baseline aerobic capacity between the two groups. As expected, our results (Figure 3) revealed a strong correlation between the baseline aerobic capacity and the 13-km finish time in the control group (*r* = −0.925, *p* < 0.001). As a result, the running time fairly reflected the aerobic performance to a certain extent. Remarkably, we further observed that DBT supplementation significantly shortened the running time with a greater impact in participants with poorer baseline aerobic capacities. Nevertheless, without a thorough examination of the physical performance, we could hardly picture all of the beneficial mechanisms of DBT on endurance and muscular fitness.

Some molecular mechanisms that have been proposed to participate in hepcidin regulation during exercise include hypoxia, erythropoiesis, circulatory iron, and inflammation [1]. In general, hepcidin levels significantly increase 0–6 h in serum and 3–24 h in urine after exercise challenge [6,9]. While it is believed that the 3-h post-exercise was the most promising time point to observe the hepcidin elevation, we collected the specimens at 0, 24, and 72 h post-exercise for the overall convenience of conducting venipuncture. In iron repleted individuals, exercise-induced inflammation could be the primary promoter of hepcidin activity, yet the basal iron status would supersede inflammation when iron-depleted (non-anaemic or anaemic) [61]. Our participants presented with normal/healthy basal serum ferritin levels, hence, in this instance, inflammation response could play a vital role as part of the hepcidin activator during exercise.

In the present study, the 13-km run increased the hepcidin levels, might further inhibit duodenal iron absorption, and trap intracellular iron. We observed substantial decreases in iron and ferritin levels immediately after the exercise challenge, which is consistent with the finding of a previous study [14]. DBT supplementation for 11 days repressed the participants’ hepcidin levels, thereby dramatically boosting iron levels and accelerating iron homeostasis during the recovery phase. The hepcidin-repression effects of DBT could be involved in the interruption of Janus kinase/signal transducers and activators of transcription proteins, bone morphogenic protein/small mothers against decapentaplegic protein, and extracellular signal-regulated kinase signal transduction pathways [23]. However, short-term DBT supplementation did not alter the levels of ferritin, which is an intracellular protein that reflects iron storage. Nonetheless, this study demonstrated the promising effect of DBT supplementation on iron regulation during exercise.

Exercise-induced haemolysis is primarily caused by oxidative stress. It has been reported that abundant free radical reactive oxygen species and reactive nitrogen species are produced during intense exercise and cause lipid peroxidation in RBC membranes, which initiates the haemolysis process [16]. MDA, a final product of polyunsaturated fatty acid peroxidation, is one of the most frequently used biomarkers for oxidative stress [62]. A significant elevation in MDA levels was observed at 24-h Rec in the control group, but not in the DBT group. The literature reports that DBT has a great antioxidant activity, especially the primary active antioxidant flavonoids that are present in AM [63]. Our results demonstrate that DBT may play a role in the attenuation of MDA levels; however, the evidence was not strong enough because of the non-significance of the observed group × time effect. The following major antioxidant enzymes possess these effects: SOD catalyses the dismutation of superoxide anions into oxygen and hydrogen peroxide, CAT detoxifies hydrogen peroxide into water, and GPx catalyses the reduction of hydrogen peroxide into water while using reduced glutathione. No substantial difference was noted in the activities of SOD and CAT in the DBT group when compared with those in the control group; however, the dissimilar responses in GPx levels at 72-h Rec between the two groups remained unknown. Haptoglobin is an indicator of haemolysis that binds free Hb released from RBCs and forms haptoglobin–Hb complexes. The response of haptoglobin levels was similar in the two groups. Haematuria is another indicator of haemolysis during intense sports activity. However, increased blood loss in urine is not the only consequence of haemolysis, as mechanical trauma also causes bleeding in the bladder wall during running [64]. Overall, we suggest that DBT supplementation did not exert a significant haemolysis-preventative effect.

The changes in the participants’ complete blood counts during exercise were similar to those that were reported in a study conducted in Taiwan on male 100-km ultramarathoners [64], but more slightly. For instance, increased WBC and platelet concentrations were found immediately after the exercise. Slight but significant decreases in Hb levels in the DBT group and MCV levels in the control group were identified. Although the decreases in Hb and MCV levels are commonly linked with iron deficiency anaemia, these phenomena are difficult to characterise, because the Hb level changes were small and within normal range. We propose that plasma expansion resulting in haemodilution could explain the slight drop in Hb levels. The fluid shifts from extravascular to intravascular space resulting in rising plasma volume to 10‒20% without reducing absolute cell mass [65]. The concentrations of red blood cell thereby drop owing to the hypervolemia; that is so-called “sports pseudoanaemia” or “false anaemia”.

In addition, we did not identify an anti-inflammatory and anti-fatigue effect of DBT, as proposed in previous murine studies [25,26]. The levels of both TNF-α and IL-6 increased at Post-Ex and 24-h Rec. DBT supplementation failed to alter the production of cytokines triggered by the intense exercise. Serum levels of AST, ALT, and plasma ammonia are sensitive biomarkers that reflect the occurrence of physical fatigue. The concentrations of these biomarkers would increase during or after an exercise challenge [66]. However, although previous studies have shown that treatment with both AM and AS reduced ammonia production in mice in a dose-dependent manner [25,26], such findings were not in accordance with this study.

We acknowledge certain limitations to this study. First, the relatively small sample size limits the overall strength of our findings. To develop an accurate model and investigate the responses thoroughly, intense exercise with multiple blood withdrawals was required, which made it difficult to recruit participants. Although this study was designed as a randomised controlled trial, large-scale study with cross-over design could benefit on minimising the study bias. Second, we recruited only male runners in our study. Because of sex differences, our data might not be representative of the biochemical responses in female runners.

## 5. Conclusions

This study is the first trial demonstrated the beneficial effects of DBT on iron regulation. Short-term DBT supplementation shortened the 13-km running time and repressed exercise-induced hepcidin levels, thereby boosting iron levels and accelerating iron homeostasis during the recovery phase. DBT may also play a role in the amelioration of MDA levels, but it failed to affect the activities of the antioxidant enzymes. Our results suggest that DBT could be a promising ergogenic aid for athletic performance.

## Figures and Tables

**Table 1:** Chemical Compounds Studied in this Article.

Compound	PubChem CID
d-Glucose	5793
Rutin	5280805
Astragaloside IV	158694
Ferulic acid	445858
Ligustilide	5319022
*n*-Butylidenephthalide	642376

**Table 2:** Characteristics of participants.

Parameter	Control (*n* = 14)	DBT (*n* = 14)
Age (year)	23.1 (2.5)	22.7 (2.0)
Height (cm)	172.4 (3.7)	173.1 (5.6)
Weight (kg)	73.3 (10.7)	69 (8.6)
HRmax (beat·min^−1^)	194.3 (10.6)	192.2 (7.3)
VO_2_max (mL·kg^−1^·min^−1^)	56.6 (11.5)	57.0 (10.6)
Finish time (min)	87.6 (15.6)	75.3 (11.3) *

Data are expressed as the mean (±standard deviation). * *p* < 0.050 compared with the control group by independent *t*-test. HRmax; maximal heart rate, VO_2_max; maximal oxygen consumption.

**Table 3:** Basal iron status of participants.

Parameter	Control (*n* = 14)	DBT (*n* = 14)
Hepcidin (ng·mL^−1^)	14.4 (7.9)	13.1 (8.2)
Iron (μg·dL^−1^)	77.4 (23.3)	99.3 (33.9)
Ferritin (ng·mL^−1^)	113.4 (96.0)	156.9 (137.7)

Data are expressed as the mean (±standard deviation).

**Table 4:** Complete blood counts and concentrations of inflammatory and metabolic markers.

Parameter	Control (*n* = 14)				DBT (*n* = 14)				Normal Range
	Pre-Tre	Post-Ex	24-h Rec	72-h Rec	Pre-Tre	Post-Ex	24-h Rec	72-h Rec	
Complete blood counts									
WBC (×10^3^·μL^−1^)	6.55 (1.66)	9.49 (1.79) ***	6.24 (1.49)	6.55 (2.15)	6.27 (1.29)	9.81 (3.20) ***	6.16 (1.43)	5.90 (1.13)	3.5–11
RBC (×10^6^·μL^−1^)	5.31 (0.29)	5.33 (0.31)	5.08 (0.34) **	5.07 (0.26) **	5.35 (0.54)	5.41 (0.71)	5.13 (0.61) **	5.09 (0.57) **	4.0–6.0
Hb (g·dL^−1^)	15.2 (0.9)	15.3 (1.0)	14.9 (0.9)	15.0 (0.7)	15.2 (0.7)	15.2 (1.1)	14.8 (0.9) *	14.7 (0.7) **	12–16
HCT (%)	47.8 (1.9)	47.7 (2.0)	45.4 (2.1) **	45.3 (1.3) **	47.4 (2.0)	47.9 (3.2)	45.4 (2.3) **	45.1 (1.9) **	36–46
MCV (fL)	90.1 (2.7)	89.7 (2.8) **	89.7 (2.8) **	89.6 (2.8) **	89.2 (7.6)	89.3 (7.3)	89.3 (7.4)	89.2 (7.3)	80–102
MCH (pg)	28.7 (1.5)	28.7 (1.1)	29.5 (1.3) **	29.6 (1.1) **	28.7 (2.7)	28.4 (2.7) *	29.1 (2.9) *	29.1 (2.8) *	27–34
MCHC (%)	31.8 (0.9)	31.9 (0.9)	32.9 (0.8) ***	33.0 (0.9) ***	32.2 (0.9)	31.8 (0.9)	32.6 (1.2)	32.6 (0.8) *	30–36
Platelet (×10^3^·μL^−1^)	253.3 (60.6)	288.3 (63.7) **	248 (50.0)	245.9 (55.0)	223.3 (43.6)	284.6 (53.9) ***	235.1 (42.9)	239.6 (49.7)	150–450
Inflammatory markers									
TNF-α (pg·mL^−1^)	2.65 (1.07)	8.79 (0.77) ***	7.72 (1.53) ***	4.36 (5.57)	3.56 (1.97)	9.54 (1.51) ***	8.09 (0.96) ***	3.33 (2.17)	
IL-6 (pg·mL^−1^)	1.10 (0.65)	9.89 (8.79) **	3.01 (0.61) ***	1.39 (0.78)	1.04 (0.44)	8.43 (4.34) ***	3.01 (0.63) ***	1.56 (0.96)	
Metabolic markers									
AST (U·L^−1^)	28.6 (33.8)	34.5 (35.7) **	30.43 (32.1)	35.4 (36.9) **	22.1 (8.8)	25.8 (12.3) *	19.6 (14.1)	26.9 (17.7)	8–38
ALT (U·L^−1^)	31.4 (46.2)	31.5 (42.7)	20.3 (27.8)	32.2 (41.5)	23.1 (28.3)	27.1 (34.5) *	16.4 (25.5) ***	28.4 (42.7)	4–40
LDH (U·L^−1^)	157.5 (17.6)	213.1 (34.7) ***	121.6 (50.2) *	185.4 (24.7) ***	167.6 (23.8)	214.4 (35.6) ***	110.1 (32.4) ***	188.6 (31.1) **	106–211
Ammonia (μg·dL^−1^)	68.1 (15.0)	159.4 (53.0) ***	62.5 (12.5)	76.3 (13.4) *	68.0 (11.4)	175.1 (46.5) ***	69.5 (15.2)	78.3 (17.9)	31–123

Data are expressed as the mean (±standard deviation). * *p* < 0.050, ** *p* < 0.010, *** *p* < 0.001 compared with Pre-Tre in each independent group using paired *t*-test. WBC; white blood cell, RBC; red blood cell, Hb; haemoglobin, HCT; haematocrit, MCV; mean corpuscular volume, MCH; mean corpuscular haemoglobin, MCHC; mean corpuscular haemoglobin concentration, TNF; tumor necrosis factor, IL; interleukin, AST; aspartate aminotransferase, ALT; alanine aminotransferase, LDH; lactic dehydrogenase.

**Table 5:** Urine OB results.

Urine OB	Control (*n* = 14)				DBT (*n* = 14)			
	Pre-Tre	Post-Ex	24-h Rec	72-h Rec	Pre-Tre	Post-Ex	24-h Rec	72-h Rec
−	13	11	12	14	14	11	14	14
+/−	1	2	2	0	0	0	0	0
+	0	1	0	0	0	0	0	0
++	0	0	0	0	0	2	0	0
+++	0	0	0	0	0	1	0	0
++++	0	0	0	0	0	0	0	0

+; positive, −; negative.

## Abbreviations

ALT	alanine aminotransferase
AM	the roots of *Astragalus membranaceus* (Fisch.) Bge. var. *mongholicus* (Bge.) Hsiao
ANOVA	analysis of variance
AS	the roots of *Angelica sinensis* (Oliv) Diels
AST	aspartate aminotransferase
CAT	catalase
DBT	Danggui Buxue Tang
ELSD	evaporative light scattering detector
GPx	glutathione peroxidase
Hb	haemoglobin
HCT	haematocrit
HPLC	high-performance liquid chromatography
HRmax	maximal heart rate
IL	interleukin
LDH	lactic dehydrogenase
MCH	mean corpuscular haemoglobin
MCHC	mean corpuscular haemoglobin concentration
MCV	mean corpuscular volume
MDA	malondialdehyde
OB	occult blood
OCP	oral contraceptive pill
PDA	photodiode array
RBC	red blood cell
RPE	rating of perceived exertion
SOD	superoxide dismutase
TNF	tumour necrosis factor
VO_2_max	maximal oxygen consumption
WBC	white blood cell

## References

[1] Kong W.N., Gao G., Chang Y.Z. (2014). Hepcidin and sports anemia. Cell Biosci..

[2] DellaValle D.M., Haas J.D. (2011). Impact of iron depletion without anemia on performance in trained endurance athletes at the beginning of a training season: A study of female collegiate rowers. Int. J. Sport Nutr. Exerc. Metab..

[3] Reinke S., Taylor W.R., Duda G.N., von Haehling S., Reinke P., Volk H.D., Anker S.D., Doehner W. (2012). Absolute and functional iron deficiency in professional athletes during training and recovery. Int. J. Cardiol..

[4] Sinclair L.M., Hinton P.S. (2005). Prevalence of iron deficiency with and without anemia in recreationally active men and women. J. Am. Diet. Assoc..

[5] Beard J., Tobin B. (2000). Iron status and exercise. Am. J. Clin. Nutr..

[6] Peeling P. (2010). Exercise as a mediator of hepcidin activity in athletes. Eur. J. Appl. Physiol..

[7] Ganz T. (2011). Hepcidin and iron regulation, 10 years later. Blood.

[8] Nemeth E., Tuttle M.S., Powelson J., Vaughn M.B., Donovan A., Ward D.M., Ganz T., Kaplan J. (2004). Hepcidin regulates cellular iron efflux by binding to ferroportin and inducing its internalization. Science.

[9] Domínguez R., Sánchez-Oliver A.J., Mata-Ordoñez F., Feria-Madueño A., Grimaldi-Puyana M., López-Samanes Á., Pérez-López A. (2018). Effects of an Acute Exercise Bout on Serum Hepcidin Levels. Nutrients.

[10] Roecker L., Meier-Buttermilch R., Brechtel L., Nemeth E., Ganz T. (2005). Iron-regulatory protein hepcidin is increased in female athletes after a marathon. Eur. J. Appl. Physiol..

[11] Peeling P., Dawson B., Goodman C., Landers G., Wiegerinck E.T., Swinkels D.W., Trinder D. (2009). Training surface and intensity: Inflammation, hemolysis, and hepcidin expression. Med. Sci. Sports Exerc..

[12] Pasiakos S.M., Margolis L.M., Murphy N.E., McClung H.L., Martini S., Gundersen Y., Castellani J.W., Karl J.P., Teien H.K., Madslien E.H. (2016). Effects of exercise mode, energy, and macronutrient interventions on inflammation during military training. Physiol. Rep..

[13] Dzedzej A., Ignatiuk W., Jaworska J., Grzywacz T., Lipinska P., Antosiewicz J., Korek A., Ziemann E. (2016). The effect of the competitive season in professional basketball on inflammation and iron metabolism. Biol. Sport.

[14] Skarpanska-Stejnborn A., Basta P., Trzeciak J., Szczesniak-Pilaczynska L. (2015). Effect of intense physical exercise on hepcidin levels and selected parameters of iron metabolism in rowing athletes. Eur. J. Appl. Physiol..

[15] Sim M., Dawson B., Landers G.J., Swinkels D.W., Tjalsma H., Wiegerinck E.T., Trinder D., Peeling P. (2014). A seven day running training period increases basal urinary hepcidin levels as compared to cycling. J. Int. Soc. Sports Nutr..

[16] Bonilla J.F., Narváez R., Chuaire L. (2005). Sports as a cause of oxidative stress and hemolysis. Colombia Médica.

[17] Lukaski H.C. (2004). Vitamin and mineral status: Effects on physical performance. Nutrition.

[18] McClung J.P., Karl J.P., Cable S.J., Williams K.W., Young A.J., Lieberman H.R. (2009). Longitudinal decrements in iron status during military training in female soldiers. Br. J. Nutr..

[19] Tolkien Z., Stecher L., Mander A.P., Pereira D.I., Powell J.J. (2015). Ferrous sulfate supplementation causes significant gastrointestinal side-effects in adults: A systematic review and meta-analysis. PLoS ONE.

[20] Wang K.P., Zeng F., Liu J.Y., Guo D., Zhang Y. (2011). Inhibitory effect of polysaccharides isolated from angelica sinensis on hepcidin expression. J. Ethnopharmacol..

[21] Liu J.Y., Zhang Y., You R.X., Zeng F., Guo D., Wang K.P. (2012). Polysaccharide isolated from angelica sinensis inhibits hepcidin expression in rats with iron deficiency anemia. J. Med. Food.

[22] Zhang Y., Li M.M., Zeng F., Yao C., Wang K.P. (2012). Study to establish the role of jak2 and smad1/5/8 pathways in the inhibition of hepcidin by polysaccharides from angelica sinensis. J. Ethnopharmacol..

[23] Zhang Y., Cheng Y., Wang N., Zhang Q., Wang K. (2014). The action of jak, smad and erk signal pathways on hepcidin suppression by polysaccharides from angelica sinensis in rats with iron deficiency anemia. Food Funct..

[24] Huang G.C., Chen S.Y., Tsai P.W., Ganzon J.G., Lee C.J., Shiah H.S., Wang C.C. (2016). Effects of dang-gui-bu-xue-tang, an herbal decoction, on iron uptake in iron-deficient anemia. Drug Des. Dev. Ther..

[25] Yeh T.S., Chuang H.L., Huang W.C., Chen Y.M., Huang C.C., Hsu M.C. (2014). Astragalus membranaceus improves exercise performance and ameliorates exercise-induced fatigue in trained mice. Molecules.

[26] Yeh T.S., Huang C.C., Chuang H.L., Hsu M.C. (2014). Angelica sinensis improves exercise performance and protects against physical fatigue in trained mice. Molecules.

[27] Yeh T.S., Hsu C.C., Yang S.C., Hsu M.C., Liu J.F. (2014). Angelica sinensis promotes myotube hypertrophy through the pi3k/akt/mtor pathway. BMC Complement. Altern. Med..

[28] Chang C.W., Chen Y.M., Hsu Y.J., Huang C.C., Wu Y.T., Hsu M.C. (2016). Protective effects of the roots of angelica sinensis on strenuous exercise-induced sports anemia in rats. J. Ethnopharmacol..

[29] Liu Y., Zhang H.G., Li X.H. (2011). A chinese herbal decoction, danggui buxue tang, improves chronic fatigue syndrome induced by food restriction and forced swimming in rats. Phytother. Res. PTR.

[30] Chinese Pharmacopoeia Commission (2015). Pharmacopoeia of the People’s Republic of China.

[31] Heyward V.H., Gibson A. (2014). Advanced Fitness Assessment and Exercise Prescription.

[32] Bruce R.A., Kusumi F., Hosmer D. (1973). Maximal oxygen intake and nomographic assessment of functional aerobic impairment in cardiovascular disease. Am. Heart J..

[33] Lee C.L., Hsu M., Astorino T.A., Liu T.W., Chang W.D. (2017). Effectiveness of 2 weeks of high-intensity interval training on performance and hormone status in adolescent triathletes. J. Sports Med. Phys. Fit..

[34] Borg G. (1998). Borg’s Perceived Exertion and Pain Scales.

[35] Gong A.G., Li N., Lau K.M., Lee P.S., Yan L., Xu M.L., Lam C.T., Kong A.Y., Lin H.Q., Dong T.T. (2015). Calycosin orchestrates the functions of danggui buxue tang, a chinese herbal decoction composing of astragali radix and angelica sinensis radix: An evaluation by using calycosin-knock out herbal extract. J. Ethnopharmacol..

[36] Zheng K.Y., Choi R.C., Guo A.J., Bi C.W., Zhu K.Y., Du C.Y., Zhang Z.X., Lau D.T., Dong T.T., Tsim K.W. (2012). The membrane permeability of astragali radix-derived formononetin and calycosin is increased by angelicae sinensis radix in caco-2 cells: A synergistic action of an ancient herbal decoction danggui buxue tang. J. Pharm. Biomed. Anal..

[37] Zheng K.Y., Zhang Z.X., Du C.Y., Zhang W.L., Bi C.W., Choi R.C., Dong T.T., Tsim K.W. (2014). Ferulic acid enhances the chemical and biological properties of astragali radix: A stimulator for danggui buxue tang, an ancient chinese herbal decoction. Planta Med..

[38] Zheng Y.Z., Choi R.C., Li J., Xie H.Q., Cheung A.W., Duan R., Guo A.J., Zhu J.T., Chen V.P., Bi C.W. (2010). Ligustilide suppresses the biological properties of danggui buxue tang: A chinese herbal decoction composed of radix astragali and radix angelica sinensis. Planta Med..

[39] Zhan J.Y., Zheng K.Y., Zhang W.L., Chen J.P., Yao P., Bi C.W., Dong T.T., Tsim K.W. (2014). Identification of angelica oil as a suppressor for the biological properties of danggui buxue tang: A chinese herbal decoction composes of astragali radix and angelica sinensis radix. J. Ethnopharmacol..

[40] Zhang Y.W., Xie D., Xia B., Zhen R.T., Liu I.M., Cheng J.T. (2006). Suppression of transforming growth factor-beta1 gene expression by danggui buxue tang, a traditional chinese herbal preparation, in retarding the progress of renal damage in streptozotocin-induced diabetic rats. Horm. Metab. Res..

[41] Lv J., Zhao Z., Chen Y., Wang Q., Tao Y., Yang L., Fan T.P., Liu C. (2012). The chinese herbal decoction danggui buxue tang inhibits angiogenesis in a rat model of liver fibrosis. Evid. Based Complement. Altern. Med..

[42] Wang P., Liang Y.Z. (2010). Chemical composition and inhibitory effect on hepatic fibrosis of danggui buxue decoction. Fitoterapia.

[43] Mak D.H., Chiu P.Y., Dong T.T., Tsim K.W., Ko K.M. (2006). Dang-gui buxue tang produces a more potent cardioprotective effect than its component herb extracts and enhances glutathione status in rat heart mitochondria and erythrocytes. Phytother. Res..

[44] Gao J., Feng L.J., Huang Y., Li P., Xu D.J., Li J., Wu Q. (2012). Total glucosides of danggui buxue tang attenuates bleomycin-induced pulmonary fibrosis via inhibition of extracellular matrix remodelling. J. Pharm. Pharmacol..

[45] Zhao P., Zhou W.C., Li D.L., Mo X.T., Xu L., Li L.C., Cui W.H., Gao J. (2015). Total glucosides of danggui buxue tang attenuate blm-induced pulmonary fibrosis via regulating oxidative stress by inhibiting nox4. Oxid. Med. Cell Longev..

[46] Zhang H., Chen S., Deng X., Yang X., Huang X. (2006). Danggui-buxue-tang decoction has an anti-inflammatory effect in diabetic atherosclerosis rat model. Diabetes Res. Clin. Pract..

[47] Gao Q.T., Cheung J.K., Li J., Chu G.K., Duan R., Cheung A.W., Zhao K.J., Dong T.T., Tsim K.W. (2006). A chinese herbal decoction, danggui buxue tang, prepared from radix astragali and radix angelicae sinensis stimulates the immune responses. Planta Med..

[48] Li X.T., Wang B., Li J.L., Yang R., Li S.C., Zhang M., Huang W., Cao L. (2013). Effects of dangguibuxue tang, a chinese herbal medicine, on growth performance and immune responses in broiler chicks. Biol. Res..

[49] Chiu P.Y., Leung H.Y., Siu A.H., Poon M.K., Dong T.T., Tsim K.W., Ko K.M. (2007). Dang-gui buxue tang protects against oxidant injury by enhancing cellular glutathione in h9c2 cells: Role of glutathione synthesis and regeneration. Planta Med..

[50] Li Y.D., Ma Y.H., Zhao J.X., Zhao X.K. (2011). Protection of ultra-filtration extract from danggui buxue decoction on oxidative damage in cardiomyocytes of neonatal rats and its mechanism. Chin. J. Integr. Med..

[51] Gao Q.T., Cheung J.K., Choi R.C., Cheung A.W., Li J., Jiang Z.Y., Duan R., Zhao K.J., Ding A.W., Dong T.T. (2008). A chinese herbal decoction prepared from radix astragali and radix angelicae sinensis induces the expression of erythropoietin in cultured hep3b cells. Planta Med..

[52] Zheng K.Y., Choi R.C., Xie H.Q., Cheung A.W., Guo A.J., Leung K.W., Chen V.P., Bi C.W., Zhu K.Y., Chan G.K. (2010). The expression of erythropoietin triggered by Danggui Buxue Tang, a Chinese herbal decoction prepared from radix astragali and radix angelicae sinensis, is mediated by the hypoxia-inducible factor in cultured hek293t cells. J. Ethnopharmacol..

[53] Haines C.J., Lam P.M., Chung T.K., Cheng K.F., Leung P.C. (2008). A randomized, double-blind, placebo-controlled study of the effect of a Chinese herbal medicine preparation (Dang Gui Buxue Tang) on menopausal symptoms in Hong Kong Chinese women. Climacteric.

[54] Wang C.C., Cheng K.F., Lo W.M., Law C., Li L., Leung P.C., Chung T.K., Haines C.J. (2013). A randomized, double-blind, multiple-dose escalation study of a chinese herbal medicine preparation (Dang Gui Buxue Tang) for moderate to severe menopausal symptoms and quality of life in postmenopausal women. Menopause.

[55] Du Q.C., Yang K.Z., Sun X.F. (2009). Efficacy of auxiliary therapy with danggui buxue decoction no.1 in treating patients of non-small cell lung cancer at peri-operational stage. Chin. J. Integr. Med..

[56] Committee on Chinese Medicine and Pharmacy (2016). Taiwan Herbal Pharmacopeia.

[57] Zhao M.M., Zhang Y., Li L.S., Yu Z.K., Li B. (2017). Efficacy and safety of danggui buxue decoction in combination with western medicine treatment of anemia for renal anemia: A systematic review and meta-analysis. Ann. Transl. Med..

[58] Xie J.H., Chen Z.W., Pan Y.W., Luo D.M., Su Z.R., Chen H.M., Qin Z., Huang S.Q., Lei G. (2016). Evaluation of safety of modified-danggui buxue tang in rodents: Immunological, toxicity and hormonal aspects. J. Ethnopharmacol..

[59] Sim M., Dawson B., Landers G., Swinkels D.W., Tjalsma H., Yeap B.B., Trinder D., Peeling P. (2015). Oral contraception does not alter typical post-exercise interleukin-6 and hepcidin levels in females. J. Sci. Med. Sport.

[60] Watts E. (1989). Athletes’ anaemia. A review of possible causes and guidelines on investigation. Br. J. Sports Med..

[61] Burden R.J., Pollock N., Whyte G.P., Richards T., Moore B., Busbridge M., Srai S.K., Otto J., Pedlar C.R. (2015). Effect of Intravenous Iron on Aerobic Capacity and Iron Metabolism in Elite Athletes. Med. Sci. Sports Exerc..

[62] Nielsen F., Mikkelsen B.B., Nielsen J.B., Andersen H.R., Grandjean P. (1997). Plasma malondialdehyde as biomarker for oxidative stress: Reference interval and effects of life-style factors. Clin. Chem..

[63] Fu J., Wang Z., Huang L., Zheng S., Wang D., Chen S., Zhang H., Yang S. (2014). Review of the botanical characteristics, phytochemistry, and pharmacology of astragalus membranaceus (huangqi). Phytother. Res..

[64] Chiu Y.H., Lai J.I., Wang S.H., How C.K., Li L.H., Kao W.F., Yang C.C., Chen R.J. (2015). Early changes of the anemia phenomenon in male 100-km ultramarathoners. J. Chin. Med. Assoc..

[65] Convertino V.A. (1991). Blood volume: Its adaptation to endurance training. Med. Sci. Sports Exerc..

[66] Chang C.-W., Huang T.-Z., Chang W.-H., Tseng Y.-C., Wu Y.-T., Hsu M.-C. (2016). Acute garcinia mangostana (mangosteen) supplementation does not alleviate physical fatigue during exercise: A randomized, double-blind, placebo-controlled, crossover trial. J. Int. Soc. Sports Nutr..

